# Association of 25-hydroxyvitamin D with HDL-cholesterol and other cardiovascular risk biomarkers in subjects with non-cardiac chest pain

**DOI:** 10.1186/s12944-019-0961-3

**Published:** 2019-01-26

**Authors:** Mohammad J. Alkhatatbeh, Noor A. Amara, Khalid K. Abdul-Razzak

**Affiliations:** Department of Clinical Pharmacy, Faculty of Pharmacy, Jordan University of Science and Technology, Irbid, 22110 Jordan

**Keywords:** Cardiovascular risk, Vitamin D, High density lipoprotein, HbA1c, Non-cardiac chest pain

## Abstract

**Background:**

Chest pain is a serious symptom that is routinely investigated as a sign of coronary artery disease. Non-cardiac chest pain (NCCP) is indistinguishable from ischemic chest pain and both are considered serious and receive similar medical investigations. Although NCCP is not associated with cardiovascular diseases (CVDs), patients with NCCP may become anxious and frightened from developing coronary events. So, it will be valuable to improve modifiable cardiovascular risk factors in such subjects to reduce fear from CVDs. Because vitamin D deficiency was considered as a possible modifiable cardiovascular risk factor, our aim was to investigate association between serum vitamin D and cardiovascular risk variables in subjects with NCCP.

**Methods:**

A cross-sectional study involved 104 subjects who underwent cardiac catheterization that did not reveal any cardiac origin for their chest pain. 25-hydroxyvitamin D was measured by electrochemiluminescence immunoassay, glucose was measured by hexokinase method, hemoglobin A1c (HbA1c) was measured by turbidimetric inhibition immunoassay and lipid profile was measured by enzymatic colorimetric assays.

**Results:**

High density lipoprotein cholesterol (HDL-C) was significantly higher in subjects with sufficient vitamin D compared to those with insufficient or deficient vitamin D (*p*-value< 0.01). 25-hydroxyvitamin D was positively associated with HDL-C (*p*-value< 0.01) and inversely associated with HbA1c (p-value = 0.02). 25-hydroxyvitamin D was not significantly correlated with other cardiovascular biomarkers including blood pressure, glucose, and other components of lipid profile (*p*-values> 0.05).

**Conclusions:**

low serum vitamin D could be involved in reducing HDL-C and increasing HbA1c and thus it may increase cardiovascular risk in subjects with NCCP.

## Introduction

Vitamin D is a steroid hormone that is initially produced in the skin after exposure to sunlight and is also obtained from dietary sources [1, 2]. It undergoes two steps of hydroxylation that occur in the liver and kidneys to produce its active form; calcitriol [3]. The traditional function of vitamin D is to maintain bone structure through its involvement in calcium and phosphate homeostasis [4]. Vitamin D increases intestinal calcium and phosphate absorption and it regulates the secretion of parathyroid hormone (PTH), which stimulates osteoclastic mobilization of calcium and phosphate from the bone [5]. Additionally, vitamin D has many other physiological functions, which are mediated by its action on vitamin D receptors (VDRs) [6]. These receptors are widely distributed across many tissues including skeletal muscle, cardiac muscle, immune cells, brain cells and cells involved in cardiovascular homeostasis [7]. So, sufficient serum vitamin D levels are required for proper bone mineralization and also for maintenance of various extra-skeletal vitamin D functions [6, 7].

Importantly, there is a growing evidence that suggests vitamin D deficiency as a novel risk factor for cardiovascular diseases (CVDs) [8]. It has been reported that vitamin D deficiency could be associated with various CVDs and their risk factors including hypertension, diabetes mellitus (DM), hyperlipidemia, heart failure and coronary artery disease [9–12]. Although mechanisms by which vitamin D deficiency can be involved in these CVDs are still not fully determined, there are some explanations for the possible cardio-protective effect of vitamin D [9]. For example, the association between vitamin D deficiency and hypertension was explained by the role of vitamin D in suppressing renin biosynthesis and thus aldosterone secretion, which regulates renal sodium excretion and blood pressure [13]. As well, vitamin D supplementation was able to inhibit ventricular remodeling in patients with heart failure [14] as vitamin D deficiency was associated with oxidative stress, cardiac inflammation, fibrosis and apoptosis [15]. Moreover, sufficient vitamin D levels may reduce atherosclerosis by decreasing macrophage cholesterol uptake and formation of foam cells [16]. VDRs are expressed in cells involved in the process of atherosclerosis including endothelial cells and vascular smooth muscle cells [17]. So, vitamin D may have a role in regulating processes that are involved in atherosclerosis such as vascular cell growth and inflammation [17].

The aim from this study was to assess the association between serum level of vitamin D and cardiovascular risk variables in subjects with non-cardiac chest pain (NCCP). These subjects are frequently present with complaints that are indistinguishable from ischemic chest pain and they receive similar medical investigations [18]. We believe that these subjects should improve their cardiovascular modifiable risk variables to reduce fear from developing acute cardiovascular events. If low serum vitamin D is associated with these risk variables, then maintenance of sufficient serum vitamin D could be valuable to reduce cardiovascular risk in this group of subjects.

## Methods

### Study design

**Fig. 1:**
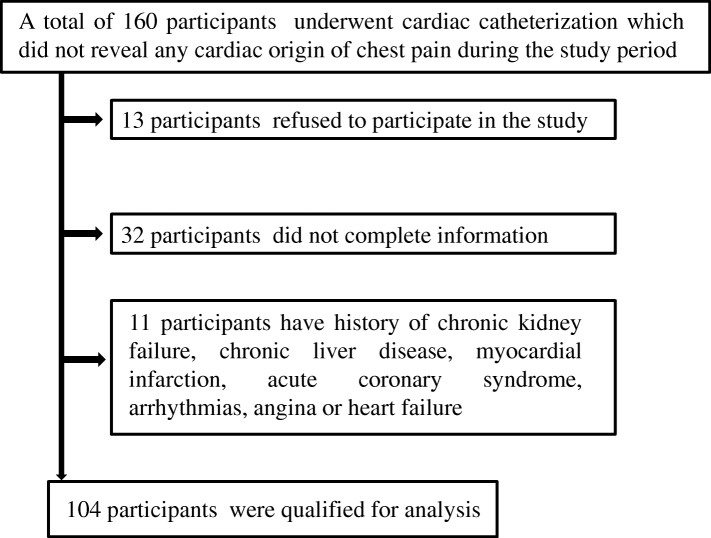
Flow chart of participant recruitment

This cross-sectional study involved 104 individuals with NCCP who were recruited from the Cardiac Catheterization Unit of King Abdullah University Hospital (KAUH; Irbid, Jordan) between May 2016 and December 2017. Figure 1 shows a flow chart involving patient selection and incorporation in this study. All participants were scheduled for a diagnostic cardiac catheterization which did not reveal any cardiac origin of their chest pain. Individuals with history of chronic kidney failure, chronic liver disease, myocardial infarction, acute coronary syndrome, arrhythmias, angina, heart failure and individuals who were supplemented with vitamin D during the previous 3 months were excluded from this study. All individuals who agreed to participate in this study had signed appropriate consent forms. The procedures of the current study were ethically approved by the Institutional Review Board (IRB) of KAUH and Jordan University of Science and Technology (Reference number 2015–531).

### Collection of data

Data about age, gender, smoking, education, recent supplementation of vitamin D and history of renal, hepatic, cardiovascular and other illnesses were collected from medical records or by self-reporting. Body mass index (BMI) was determined using the equation: BMI = body weight (Kg) ÷ squared body height (m^2^). Systolic blood pressure (SBP) and diastolic blood pressure (DBP) were measured at rest using a mercury sphygmomanometer by a well-trained registered nurse.

### Blood collection and processing

Venous blood samples were collected by a well-trained lab technician to determine levels of 25-hydroxyvitamin D, fasting blood glucose (FBG), hemoglobin A1c (HbA1c) and lipid profile. Serum was prepared by centrifuging blood samples at 2100 g for 8 min at room temperature within 1 h of blood sampling using a high speed Jouan centrifuge (Thermo Fisher Scientific, Inc., Waltham, MA, USA).

### Laboratory assays

The electrochemiluminescence immunoassay was used to measure serum concentration of 25-hydroxyvitamin D using Roche Modular E170 Analyzer (Roche Diagnostics, Basel, Switzerland). Participants with serum 25-hydroxyvitamin concentration ≥ 30 ng/mL were classified as having sufficient vitamin D level, participants with serum 25-hydroxyvitamin concentration between 20 and 30 ng/mL were classified as having insufficient vitamin D level and participants with serum 25-hydroxyvitamin concentration < 20 ng/mL were classified as having deficient vitamin D level [19]. FBG concentration was measured by the hexokinase method using Hitachi 902 auto-analyzer (Roche Diagnostics GmbH, Mannheim, Germany). HbA1c level was measured by the turbidimetric inhibition immunoassay using cobas b 101 analyzer (Roche Diagnostics GmbH, Mannheim, Germany). High density lipoprotein cholesterol (HDL-C), low density lipoprotein cholesterol (LDL-C), triglycerides (TGs) and total cholesterol were measured by enzymatic colorimetric assays using cobas c 501 analyzer (Roche Diagnostics GmbH, Mannheim, Germany). Participants were considered having dyslipidemia if they have total cholesterol > 5.18 mmol/L (*n* = 71, 68.3%), TGs > 1.70 mmol/L (n- 64, 61.5%), LDL-C > 3.37 mmol/L (*n* = 42, 40.4%) or HDL-C < 1.55 mmol/L (*n* = 76, 64.4%) as described in Ge et al., 2017 study [20].

### Statistical analysis

Data were analyzed using the IBM SPSS statistical software version 20 (Armonk, New York, USA). Variables were expressed as frequency (%), mean ± standard deviation (SD) or median (25th – 75th percentiles). Chi-square test or Fisher’s exact test was used to determine differences between categorical variables. Kruskal-Wallis H test was used to determine differences between continuous variables. Continuous variables that were not normally distributed were log transformed before correlation analysis. Correlations between continuous variables were determined by Pearson’s product-moment test. Associations between continuous variables were determined by multiple linear regression analysis. Association between serum vitamin D level groups (as dependent variable) and serum TGs, LDL-C, HDL-C and dyslipidemia level groups were determined by ordinal logistic regression analysis using two models to adjust for confounding variables. Model 1 included age, gender, education level and type of lipid as co-variables while model 2 included smoking, HbA1c and type of lipid as co-variables. Level of statistical significance was set at *p*-value < 0.05.

## Results

### General and biochemical characteristics

Data were obtained from 104 individuals (64 males and 40 females) with NCCP whom age ranged from 25 to 78 years. Participants were divided according to their vitamin D status into sufficient (6.7%), insufficient (17.3%) and deficient (76%) vitamin D levels. General and biochemical characteristics of the participants are shown in Table 1. Significant difference between participants with deficient, insufficient and sufficient vitamin D levels was detected only for HDL-C (*p*-value < 0.01).

**Table 1:** Characteristics of participants according to their vitamin D status

Variable	Total (*n* = 104)	Sufficient vitamin D status (*n* = 7)	Insufficient vitamin D status (*n* = 18)	Deficient vitamin D status (*n* = 79)	*P*-value^*^
Age (Year)					
Mean ± SD	50.77 ± 11.03	55.0 ± 11.61	52.28 ± 11.89	50.05 ± 10.81	0.39
Mean rank	–	65.07	56.50	50.47	
BMI (Kg/m^2^)					0.19
Mean ± SD	30.58 ± 5.61	29.71 ± 3.68	28.56 ± 4.89	31.11 ± 5.83	
Mean rank	–	50.79	41.03	55.27	
Gender					
Male	64 (61.5)	2 (28.6)	12 (66.7)	50 (63.3)	0.18
Female	40 (38.5)	5 (71.4)	6 (33.3)	29 (36.7)	
Smoking					
Yes	42 (40.4)	4 (57.1)	6 (33.3)	32 (40.5)	0.56
No	62 (59.6)	3 (42.9)	12 (66.7)	47 (59.5)	
Education					0.94
Below high school	64 (61.5)	5 (71.4)	11 (61.1)	48 (60.8)	
High school or above	40 (38.5)	2 (28.6)	7 (38.9)	31 (39.2)	
SBP (mmHg)					
Mean ± SD	129.74 ± 13.65	131.14 ± 6.52	128.33 ± 17.82	129.94 ± 13.15	0.90
Mean rank	-	57.36	51.75	52.24	
DBP (mmHg)					
Mean ± SD	80.75 ± 9.78	84.14 ± 9.92	80.00 ± 12.37	80.62 ± 9.17	0.66
Mean rank	–	62.29	52.36	51.66	
FBG (mmol/L)					
Median (25th–75th percentiles)	6.70 (5.30–8.70)	6.60 (5.60–8.40)	6.15 (4.95–7.85)	6.80 (5.43–8.80)	0.34
Mean rank	-	49.43	40.67	51.68	
HbA1c (%)					
Median (25th–75th percentiles)	5.80 (5.50–6.87)	5.9 (4.76–6.39)	5.61 (5.30–5.79)	5.99 (5.59–7.75)	0.05
Mean rank	-	32.92	26.13	41.24	
HDL-C(mmol/L)					
Mean ± SD	1.06 ± 0.28	1.31 ± 0.15	1.17 ± 0.32	1.01 ± 0.25	< 0.01
Mean rank		81.57	60.42	45.22	
LDL-C (mmol/L)					
Mean ± SD	3.20 ± 0.95	3.65 ± 0.75	3.11 ± 0.98	3.18 ± 0.96	0.29
Mean rank	-	66.79	47.19	49.77	
TGs (mmol/L)					
Median (25th–75th percentiles)	2.04 (1.48–2.79)	1.35 (1.26–3.47)	1.74 (1.38–2.27)	2.07 (1.57–2.88)	0.14
Mean rank	-	39.29	41.14	53.79	
Total Cholesterol (mmol/L)					
Mean ± SD	4.73 ± 1.08	5.38 ± 0.88	4.67 ± 1.07	4.68 ± 1.09	0.20
Mean rank	-	69.57	49.58	48.94	
Dyslipidemia					0.35
Normal	15 (14.4)	2 (28.6)	3 (16.7)	10 (12.7)	
Dyslipidemia	89 (85.6)	5 (71.4)	15 (83.3)	69 (87.3)	
25-hydroxyvitamin D (ng/mL)					
Median (25th–75th percentiles)	12.76 (8.29–19.72)	42.0 (32.10–43.42)	22.87 (20.89–24.46)	9.85 (7.27–14.74)	< 0.001
Mean rank	-	101.00	88.50	40.00	

^*^Chi-square test or Fisher’s exact test for categorical variables and Kruskal-Wallis H test for continuous variables (*p*-value < 0.05 is considered significant). Data are expressed as frequency (%), mean ± standard deviation or median (25th–75th percentiles). BMI; body mass index, SBP; systolic blood pressure, DBP; diastolic blood pressure, HDL-C, high density lipoprotein-cholesterol, LDL-C; low density lipoprotein-cholesterol, TGs; Triglycerides; FBG; fasting blood glucose, HbA1c; hemoglobin A1c, SD; standard deviation

### Correlation of 25-hydroxyvitamin D with cardiovascular risk biomarkers

As shown in Tables 2, 25-hydroxyvitamin D was significantly inversely correlated with BMI (r = − 0.26, *p*-value < 0.01) and HbA1c (r = − 0.29, p-value = 0.01) and directly correlated with HDL-C (r = 0.23, p-value = 0.02). In contrast, 25-hydroxyvitamin D was not significantly correlated with age, SBP, DBP, FBG, LDL-C, TGs and Total Cholesterol (*p*-values > 0.05).

**Table 2:** Correlation of 25-hydroxyvitamin D with cardiovascular risk biomarkers

	BMI (Kg/m^2^)	SBP (mmHg)	DBP (mmHg)	Log (FBG (mmol/L))	Log (HbA1c (%))	HDL-C (mmol/L)	LDL-C (mmol/L)	Log (TGs (mmol/L))	Total Cholesterol (mmol/L)	Log (25-hydroxyvitamin D (ng/mL))
Age (years)	r = 0.05 p = 0.62	r = 0.08 p = 0.43	r = 0.02 *p* = 0.83	r = 0.20 *p* = 0.05	r = 0.26 p = 0.02	r = 0.14 *p* = 0.17	r = − 0.10 *p* = 0.33	r = − 0.09 *p* = 0.35	r = − 0.07 p = 0.48	r = 0.01 *p* = 0.94
BMI (Kg/m^2^)	**–**	r = 0.07 *p* = 0.47	r = 0.25 *p* = 0.01	r = − 0.03 *p* = 0.80	r = − 0.01 *p* = 0.91	r = − 0.06 *p* = 0.54	r = − 0.07 *p* = 0.51	r = 0.10 *p* = 0.31	r = − 0.05 *p* = 0.62	r = − 0.26 *p* < 0.01
SBP (mmHg)	**–**	**–**	r = 0.70 *p* < 0.001	r = 0.04 *p* = 0.68	r = 0.15 *p* = 0.19	r = 0.02 *p* = 0.87	r = 0.07 *p* = 0.52	r = 0.05 p = 0.62	r = 0.07 *p* = 0.48	r = − 0.14 *p* = 0.15
DBP (mmHg)	**–**	**–**	**–**	r = 0.12 *p* = 0.23	r = 0.03 *p* = 0.79	r = 0.11 *p* = 0.27	r = 0.04 *p* = 0.70	r = − 0.08 *p* = 0.43	r = 0.05 *p* = 0.61	r = − 0.06 *p* = 0.55
Log (FBG (mmol/L))	**–**	**–**	**–**	**–**	r = 0.72 *p* < 0.001	r = − 0.18 *p* = 0.13	r = 0.09 *p* = 0.38	r = 0.14 *p* = 0.18	r = 0.10 *p* = 0.36	r = − 0.12 *p* = 0.24
Log (HbA1c (%))	**–**	**–**	**–**	**–**	**–**	r = − 0.18 p = 0.13	r = 0.04 *p* = 0.76	r = 0.28 *p* = 0.02	r = 0.04 *p* = 0.72	r = − 0.29 *p* = 0.01
HDL-C (mmol/L)	**–**	**–**	**–**	**–**	**–**	**–**	r = 0.21 *p* = 0.04	r = − 0.30 *p* < 0.01	r = 0.39 p < 0.001	r = 0.23 p = 0.02
LDL-C (mmol/L)	**–**	**–**	**–**	**–**	**–**	**–**	**–**	r = 0.13 *p* = 0.20	r = 0.96 p < 0.001	r = 0.14 *p* = 0.16
Log (TGs (mmol/L))	**–**	**–**	**–**	**–**	**–**	**–**	**–**	**–**	r = 0.25 p = 0.01	r = − 0.12 p = 0.24
Total Cholesterol (mmol/L)	**–**	**–**	**–**	**–**	**–**	**–**	**–**	**–**	**–**	r = 0.17 *p* = 0.09

Pearson product-moment test (*p*-value < 0.05 is considered significant). *BMI* body mass index, *SBP* systolic blood pressure, *DBP* diastolic blood pressure, *HDL-C* high density lipoprotein-cholesterol, *LDL-C* low density lipoprotein-cholesterol, *TGs* Triglycerides. *FBG* fasting blood glucose, *HbA1c* hemoglobin A1c, r; Pearson’s correlation coefficient

### Prediction of cardiovascular risk variables and serum 25-hydroxyvitamin D

Multiple linear regression analyses were performed to determine predictors of cardiovascular risk variables and serum 25-hydroxyvitamin D. Table 3 shows that 25-hydroxyvitamin D was significantly associated with HDL-C (*p*-value < 0.01) and negatively associated with HbA1c (*p*-value = 0.02). SBP was significantly associated with DBP (*p*-value < 0.01) and DBP was significantly associated with BMI (*p*-value = 0.02). FBG was significantly associated with HbA1c (*p*-value < 0.001). HDL-C was significantly associated with LDL-C (p-value < 0.01), 25-hydroxyvitamin D (p-value = 0.02) and gender (p-value < 0.01) and negatively associated with TGs (p-value < 0.01). TGs level was associated with HbA1c (p-value < 0.05) and negatively associated with HDL-C (p-value = 0.03). Total cholesterol was associated with HDL-C, LDL-C and TGs (*p*-values < 0.001). Further ordinal logistic regression analyses were performed to find association between vitamin D level groups and serum lipid levels (TGs, HDL-C and LDL-C) using model 1 and model 2 as described in methods. As shown in Table 4, HDL-C was significantly associated with serum vitamin D in both models (Model 1 odds ratio (confidence interval) was 0.34 (0.12–0.92), *p*-value = 0.04 and model 2 odds ratio (confidence interval) was 0.32 (0.11–0.98), p-value < 0.05). In contrast, there was no significant association between vitamin D level groups and either TGs or LDL-C levels using both models. In addition, there was no significant association between vitamin D level groups and dyslipidemia groups (Table 5), which were defined according to any abnormal level of any component of the lipid profile as described in methods.

**Table 3:** Prediction of cardiovascular risk variables and serum 25-hydroxyvitamin D

Variables	R^2^	ANOVA	Model	B	β	P-value^*^
Log (25-hydroxyvitamin D (ng/mL))	0.26	F = 4.54, p-value < 0.01	Constant BMI HDL-C Log (HbA1c) Gender Smoking	1.55 <− 0.01 0.38 − 0.69 − 0.03 − 0.08	- −0.05 0.34 − 0.25 − 0.06 − 0.24	< 0.001 0.69 < 0.01 0.02 0.69 0.09
SBP (mmHg)	0.51	F = 34.0, *p*-value < 0.001	Constant DBP Gender Smoking	52.16 1.00 −3.31 0.61	- 0.72 0.72 0.04	< 0.01 < 0.01 0.16 0.62
DBP (mmHg)	0.55	F = 30.13, p-value < 0.001	Constant BMI SBP Gender Smoking	3.99 0.32 0.49 2.65 − 0.42	- 0.18 0.69 0.13 − 0.04	0.58 0.02 < 0.001 0.10 0.65
Log (FBG (mmol/L))	0.54	F = 18.98, p-value < 0.001	Constant Age Log (HbA1c) Gender Smoking	− 0.10 < 0.01 1.14 0.03 − 0.03	- 0.07 0.71 0.09 − 0.15	0.39 0.47 < 0.001 0.42 0.18
Log (HbA1c (%))	0.57	F = 17.48, p-value < 0.001	Constant Log (FBG) Log (TGs) Log (25-hydroxyvitamin D) Gender Smoking	0.48 0.42 0.08 − 0.06 − 0.01 0.01	- 0.67 0.14 − 0.15 − 0.04 − 0.06	< 0.001 < 0.001 0.09 0.09 0.66 0.59
HDL-C (mmol/L)	0.33	F = 9.17, p-value < 0.001	Constant LDL-C Log (TGs) Log (25-hydroxyvitamin D) Gender Smoking	0.40 0.07 − 0.34 0.21 0.20 0.02	- 0.24 − 0.24 0.22 0.36 0.07	0.01 < 0.01 < 0.01 0.02 < 0.01 0.52
LDL-C (mmol/L)	0.09	F = 3.15, p-value = 0.03	Constant HDL-C Gender Smoking	2.75 0.91 − 0.09 − 0.20	- 0.26 − 0.05 − 0.19	< 0.001 0.02 0.72 0.10
Log (TGs (mmol/L))	0.17	F = 3.42, p-value = 0.01	Constant Log (HbA1c) HDL-C Gender Smoking	0.23 0.41 −0.21 − 0.02 − 0.01	- 0.23 − 0.28 − 0.04 − 0.02	0.26 < 0.05 0.03 0.78 0.86
Total Cholesterol	1.00	F = 5240.16, p-value < 0.001	Constant HDL-C LDL-C Log (TGs) Gender Smoking	0.04 1.05 1.00 1.19 0.01 < 0.001	- 0.27 0.88 0.22 < 0.01 < 0.001	0.25 < 0.001 < 0.001 < 0.001 0.72 0.96

^*^Multiple linear regression analysis (p-value < 0.05 is considered significant). *R*^*2*^ squared coefficient of determination, B; unstandardized coefficient, β; standardized coefficient; F, F-statistic, SBP; systolic blood pressure, DBP; diastolic blood pressure, BMI; body mass index, HbA1c; hemoglobin A1c, FBG; fasting blood glucose, HDL-C, high density lipoprotein-cholesterol, *LDL-C* low density lipoprotein-cholesterol, *TGs* Triglycerides

**Table 4:** Ordinal logistic regression analysis for serum lipid levels and serum vitamin D level groups

Estimate Standard error Wald Odd ratio (95% confidence interval) P-value*					
Triglycerides (TGs)					
Model 1	Estimate	Standard error	Wald	Odd ratio (95% confidence interval)	P-value^*^
Vitamin D status 1	1.99	1.31	2.31	7.34 (0.56–96.04)	0.13
Vitamin D status 2	3.52	1.36	6.71	33.70 (2.35–482.75)	0.01
High TGs Normal TGs (Ref.)	−0.77	0.47	2.67	0.46 (0.18–1.17)	0.10
Age	0.03	0.02	1.28	1.03 (0.98–1.07)	0.26
Male gender Female gender (Ref.)	−0.11	0.49	0.05	0.90 (0.34–2.34)	0.82
Education:					
Below high school High school or above (Ref.)	0.14	0.49	0.08	1.15 (0.44–2.97)	0.78
Model 2					
Vitamin D status 1	− 3.29	1.94	2.88	0.04 (0.001–1.67)	0.09
Vitamin D status 2	−1.66	1.94	0.73	0.19 (0.004–8.52)	0.39
High TGs Normal TGs (Ref.)	−0.78	0.55	2.01	0.46 (0.16–1.35)	0.16
Smoking Non-smoking (Ref.)	−0.17	0.59	0.09	0.84 (0.27–2.65)	0.77
HbA1c	−0.61	0.33	3.55	0.54 (0.29–1.03)	0.06
High density lipoprotein cholesterol (HDL-C)					
Model 1					
Vitamin D status 1	2.19	1.39	2.50	8.92 (0.59–134.67)	0.11
Vitamin D status 2	3.73	1.43	6.81	41.66 (2.53–685.79)	0.01
Low HDL-C Normal HDL-C (Ref.)	−1.09	0.52	4.47	0.34 (0.12–0.92)	0.04
Age	0.03	0.02	1.44	1.03 (0.98–1.08)	0.23
Male gender Female gender (Ref.)	0.19	0.54	0.13	1.21 (0.42–3.49)	0.72
Education:					
Below high school High school or above (Ref.)	0.25	0.49	0.25	1.28 (0.49–3.37)	0.62
Model 2					
Vitamin D status 1	−3.80	2.03	3.50	0.02 (0.00–1.20)	0.06
Vitamin D status 2	−2.13	2.02	1.12	0.12 (0.00–6.20)	0.29
Low HDL-C Normal HDL-C (Ref.)	−1.13	0.56	4.02	0.32 (0.11–0.98)	< 0.05
Smoking Non-smoking (Ref.)	0.03	0.59	0.00	1.03 (0.32–3.31)	0.96
HbA1c	−0.67	0.33	3.99	0.51 (0.27–0.99)	< 0.05
Low density lipoprotein cholesterol (LDL-C)					
Model 1					
Vitamin D status 1	2.41	1.35	3.17	11.01 (0.78–156.91)	0.08
Vitamin D status 2	3.91	1.40	7.81	49.81 (3.21–772.72)	0.01
High LDL-C Normal LDL (Ref.)	0.31	0.47	0.44	1.36 (0.55–3.38)	0.51
Age	0.02	0.02	1.16	1.02 (0.98–1.07)	0.28
Male gender Female gender (Ref.)	−0.28	0.49	0.34	0.75 (0.29–1.95)	0.56
Education:					
Below high school High school or above (Ref.)	0.12	0.48	0.06	1.13 (0.44–2.90)	0.80
Model 2					
Vitamin D status 1	−3.38	2.03	2.79	0.03 (0.00–1.80)	0.10
Vitamin D status 2	−1.80	2.03	0.78	0.17 (0.00–8.88)	0.38
High LDL-C Normal LDL-C (Ref.)	0.19	0.56	0.11	1.21 (0.40–3.62)	0.74
Smoking Non-smoking (Ref.)	−0.22	0.58	0.15	0.80 (0.26–2.47)	0.70
HbA1c	−0.71	0.34	4.24	0.49 (0.25–0.97)	0.04

*Ordinal logistic regression analysis (ordinal dependent variable was vitamin D status: deficient, insufficient and sufficient vitamin D levels in order). We used two models of logistic regression using two groups of co-variables. Model 1 included age, gender, education level and type of lipid as co-variables while model 2 included smoking, HbA1c and type of lipid as co-variables. P-values < 0.05 were considered statistically significant. *TGs* triglycerides, *HDL-C* high density lipoprotein cholesterol, *LDL-C* low density lipoprotein cholesterol, *HbA1c* hemoglobin A1c

**Table 5:** Ordinal logistic regression analysis for dyslipidemia and serum vitamin D level groups

Model 1	Estimate	Standard error	Wald	Odd ratio (95% confidence interval)	P-value^*^
Vitamin D status 1	1.97	1.36	2.10	7.15 (0.50-102.01)	0.15
Vitamin D status 2	3.47	1.40	6.16	32.21 (2.08-499.97)	0.01
Dyslipidemia Normal lipids (Ref.)	-0.54	0.62	0.74	0.59 (0.17-1.99)	0.39
Age	0.03	0.02	1.27	1.03 (0.98-1.07)	0.26
Male gender Female gender (Ref.)	-013	0.51	0.07	0.88 (0.33-2.36)	0.79
Education Below high school High school or above (Ref.)	0.11	0.49	0.06	1.12 (0.43-2.90)	0.82
Model 2					
Vitamin D status 1	-3.44	1.93	3.18	0.03 (0.00-1.41)	0.08
Vitamin D status 2	-1.84	1.93	0.91	0.16 (0.00-6.96)	0.34
Dyslipidemia Normal lipids (Ref.)	-0.51	0.67	0.58	0.60 (0.16-2.24)	0.45
Smoking Non-smoking (Ref.)	-0.16	0.58	0.08	0.85 (0.27-2.64)	0.78
HbA1c	-064	0.33	3.84	0.53 (0.28-1.00)	0.05

* Ordinal logistic regression analysis (ordinal dependent variable was vitamin D status: deficient, insufficient and sufficient vitamin D levels in order). We used two models of logistic regression using two groups of co-variables. Model 1 included age, gender, education level and type of lipid as co-variables while model 2 included smoking, HbA1c and type of lipid as co-variables. P-values < 0.05 were considered statistically significant. TGs; triglycerides, HDL-C; high density lipoprotein cholesterol, LDL-C; low density lipoprotein cholesterol, HbA1c; hemoglobin A1c.

## Discussion

Chest pain is a serious warning symptom that is routinely investigated as a sign of coronary artery disease [21]. However, a large percent (> 60%) of chest pain presentations is found to be of non-cardiac origin [22]. Although NCCP is not associated with CVDs, patients with chronic NCCP may become anxious and afraid of developing life-threatening cardiovascular events as NCCP is indistinguishable from ischemic chest pain and as they may coexist simultaneously [23, 24]. So, we believe that it will be valuable to reduce cardiovascular risk among such patients by improving their cardiovascular modifiable risk factors. This will reduce the opportunity to develop CVDs and improve their quality of life by reducing fear from cardiovascular events. Because vitamin D has been reported as a possible cardio-protective vitamin [9] and vitamin D deficiency as a potential modifiable cardiovascular risk factor [25], the current study was interested to find association between serum vitamin D and the classical cardiovascular risk variables in adult subjects with NCCP.

This study has shown that the only cardiovascular variable that was significantly different between participants with sufficient, insufficient and deficient vitamin D levels was the HDL-C (Table 1). Participants with sufficient vitamin D levels were having higher HDL-C compared to participants with insufficient and sufficient vitamin D levels. Correlation and regression analyses have also shown significant positive association between serum 25-hydroxyvitamin D and HDL-C levels (Tables 2, 3 and 4). So, higher vitamin D levels may result in increased HDL-C and thus decreased cardiovascular risk. Additionally, the current study did not show any correlation between serum 25-hydroxyvitamin D and other components of the lipid profile including LDL-C, TGs and total cholesterol. As well, this study did not show any association between vitamin D level groups and dyslipidemia (defined by any abnormal component of lipid profile) groups (Table 5). To the best of our knowledge, there were no previous studies that investigated the association between serum vitamin D and cardiovascular risk variables in subjects with NCCP. However, our findings were similar to results of other studies that were performed on other study groups. These include Auwerx J et al. study [26], in which vitamin D was positively correlated with HDL-C in men and women, Kazlauskaite R et al. study [27], in which vitamin D was associated HDL-C in postmenopausal women, Iqbal AM et al. study [25], in which vitamin D was associated HDL-C in obese children, Ge H et al. study [20], in which vitamin D was associated with HDL-C in rural population of china and Wang E et al. study [28], in which lower HDL-C levels were seen in subjects with plasma 25-hydroxyvitamin D < 25 nmol/L. In contrast, Black LJ et al. study [29] did not show any significant association between vitamin D and HDL-C in adolescents and young adults. Unfortunately, studies that investigated the effect of vitamin D supplementation on lipid profile did not show consistent results. For instance, Tavakoli et al. [30] have shown that vitamin D supplementation may have a positive impact on HDL-C level in healthy school children and may be effective in reducing risk of CVDs on long run. Whereas, Schwetz V et al. [31] have shown that vitamin D supplementation may have an unfavorable effect on lipid profile as it increased total cholesterol, TGs, LDL-C and HDL-C. As well, a meta-analysis that was performed by Wang H et al. [32] did not show any significant effect for vitamin D supplementation on total cholesterol, TGs or HDL-C. Even though, the association between serum vitamin D and HDL-C levels in participants with NCCP is reported here for the first time and this could encourage further research to find the effect of vitamin D supplementation on HDL-C level in this group of subjects and to check what is the mechanism by which vitamin D can affect the level of HDL-C.

Serum 25-hydroxyvitamin D was also negatively associated with HbA1c level and was not significantly correlated with FBG level in participants with NCCP (Tables 2 and 3). The difference between HbA1c and FBG measurements is that HbA1c is a measurement of glycemic control over the past 2 months while FBG is a measurement of a single blood glucose concentration after an overnight fasting [33]. This suggests that low vitamin D levels may increase risk of chronic hyperglycemia and type 2 DM, which is also considered as a risk factor for CVDs [34]. To the best of our knowledge, there were no previous studies that assessed the association between vitamin D and measures of glycemic control in subjects with NCCP or even in subjects without DM. However, the relationship between vitamin D, HbA1c and FBG in this study was similar to those reported by other studies performed in patients with DM [35, 36].

The current study did not show any correlation between serum 25-hydroxyvitamin D and either SBP or DBP in participants with NCCP (Table 2). Although vitamin D was investigated as a negative regulator of renin-angiotensin system and thus blood pressure [13], previous studies did not show any benefit from vitamin D supplementation on both renin-angiotensin system or reducing blood pressure [37–39]. So, our results were consistent with these studies and suggest that low serum vitamin D levels may not affect cardiovascular risk by increasing blood pressure.

Collectively, this study has reported for the first time a positive association between serum 25-hydroxyvitamin D and HDL-C levels and a negative association between serum 25-hydroxyvitamin D and HbA1c levels in subjects with NCCP. This suggests that low serum vitamin D could be involved in reducing HDL-C and increasing HbA1c levels and thus increases cardiovascular risk in these subjects. However, the present study has some limitations that may affect its results. The cross-sectional design of this study prevented us from extrapolating any causal association between serum vitamin D and HDL-C or HbA1c. So, we recognize that the absence of a control group and follow up with vitamin D supplementation may be reasonable limitations. As well, we were unable to increase the number of study participants because of fund limitation. Concerning study variables, we did not collect data from participants about the content of fat, fruit, vegetables, tea and alcohol drinking as suggested in Ge et al. study [20] because these may affect the lipid profile of study participants. However, our study sample was obtained from a Muslim country, in which alcohol is prohibited by religion and tea is considered as a traditional drink. Fat content in the diet and amount of fruits and vegetables will be considered for future studies.

## Conclusion

This study provides an evidence of a positive association between serum 25-hydroxyvitamin D and HDL-C levels and a negative association between serum 25-hydroxyvitamin D and HbA1c levels in subjects with NCCP. These results support the hypothesis that vitamin D could act as a cardio-protective vitamin, especially by its association with the athero-protective HDL-C. This will direct further studies to assess vitamin D supplementation on serum levels of HDL-C.

## Abbreviations

BMI: Body mass index
CVDs: Cardiovascular diseases
DBP: Diastolic blood pressure
DM: Diabetes mellitus,
FBG: Fasting blood glucose
HbA1c: Hemoglobin A1c
HDL-C: High density lipoprotein cholesterol
LDL-C: Low density lipoprotein cholesterol
NCCP: Non-cardiac chest pain
PTH: Parathyroid hormone
SBP: Systolic blood pressure
SD: Standard deviation
TGs: Triglycerides
VDRs: Vitamin D receptors
